# High-Energy Transsyndesmotic Ankle Fracture Dislocation: A Case Report and Systematic Literature Review

**DOI:** 10.1155/2018/7902641

**Published:** 2018-10-28

**Authors:** Yinshuan Deng, Chenhui Dong, Xiaojie Yang, Rui Liu, Feiyi Hou, Shensong Li, Kanglai Tang

**Affiliations:** ^1^Department of Sports Medicine, Lanzhou General Hospital of Lanzhou Military Command, Lanzhou, Gansu 730050, China; ^2^Department of Orthopaedic Surgery, Southwest Hospital, The Army Military Medical University, Chongqing, Chongqing 400038, China

## Abstract

High-energy trauma can cause transsyndesmotic ankle fracture dislocation. These fractures are quite rare. Here we present a clinical case of a male patient with this type of injury. A systematic review of PubMed, Ovid MEDLINE, and Embase electronic databases revealed only two prior publications on a similar topic. We discuss the typical clinical features of these injuries, the treatment of high-energy trauma which can cause transsyndesmotic ankle fracture dislocation, and its prognosis.

## 1. Introduction

The fractures that occur as a consequence of an external high-energy impact that leads to a transsyndesmotic injury are called high-energy transsyndesmotic ankle translocation fractures. These fractures may present with a rotational or bending element or with talus displacement into the distal tibiofibular joint. Either (or both) mechanisms cause syndesmotic displacement and soft-tissue injury. Because the action of the displaced talus resembles that of a logsplitter wedge, these types of transsyndesmotic fractures are also known as the “logsplitter” injury.

These fractures are rarely described in the literature. We are aware of only two recent publications [1, 2] that have described patients with such injury. It can therefore be stated that substantial knowledge gaps exist with regard to diagnosis and treatment of these fractures. In our recent clinical practice, we have treated a patient with a high-energy transsyndesmotic ankle translocation fracture. Subsequent to treating this patient, we wanted to better assess the available knowledge and conducted a systematic review of the literature for the past 10 years. In this report, we present the clinical case and systematic review of the literature on transsyndesmotic ankle translocation fractures.

## 2. Case Report and Presentation of the Literature

Patient permission was procured for reporting this clinical case.

**Figure 1:**
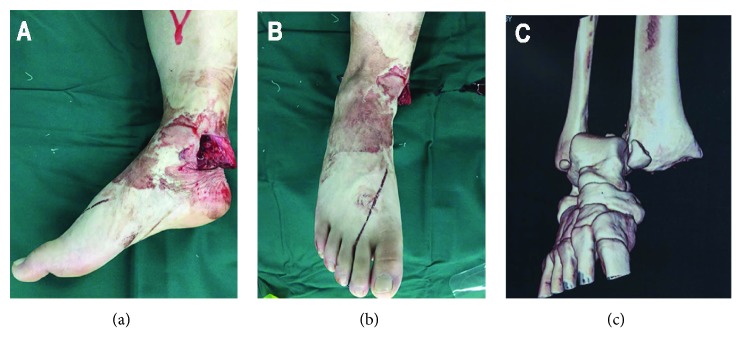
Prereduction view and CT 3D reconstruction of the ankle fracture. (a) Lateral view of the injured ankle. Note displaced distal tibia. (b) Frontal view. (c) CT 3D reconstruction. Talus “wedging” into the distal tibiofibular joint and displacement of tibia are evident.

**Figure 2:**
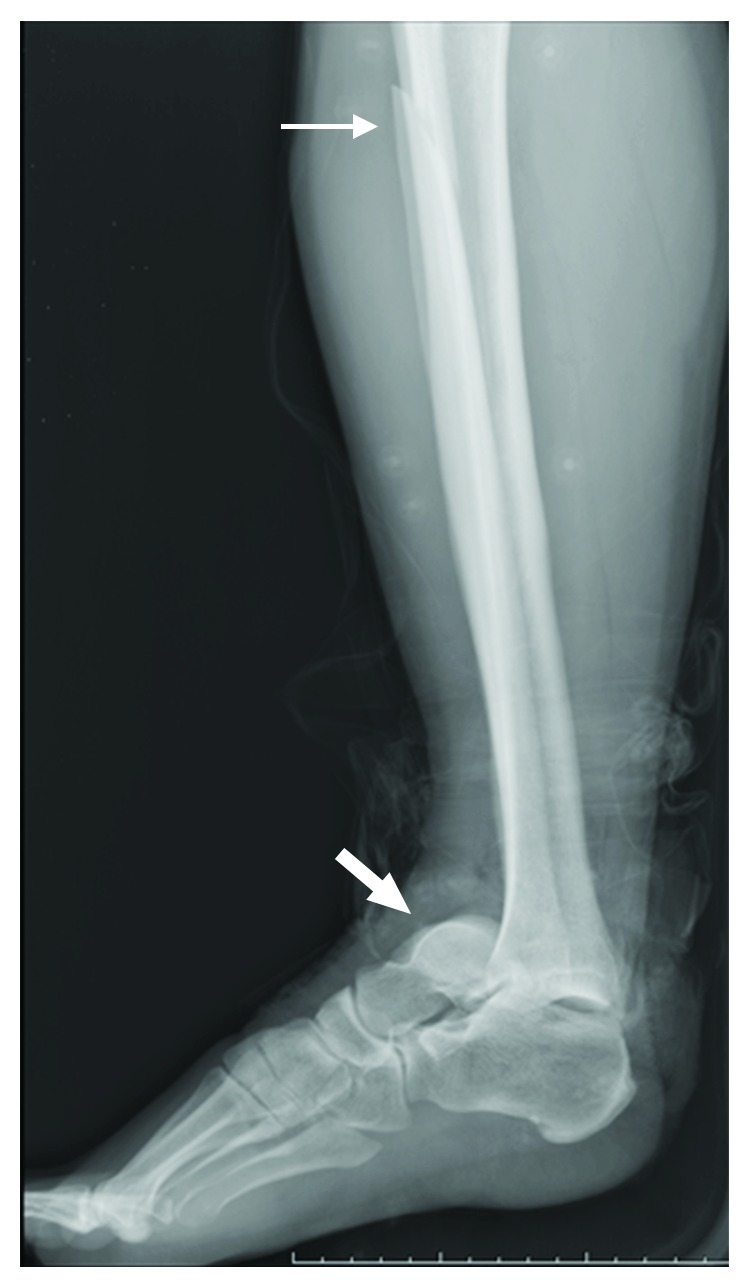
Lateral radiograph of the right shin and foot. Anterior dislocation of the talus (thick white arrow) and proximal fibular fracture (thin white arrow) are seen.

**Figure 3:**
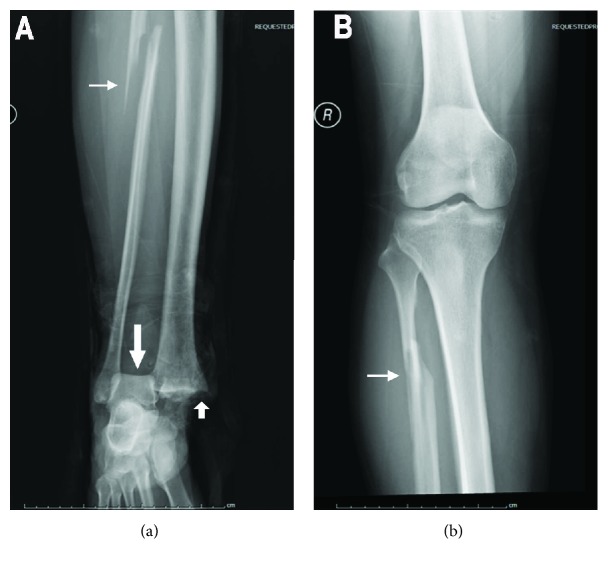
Anteroposterior radiographs of the right shin and foot and the knee joint. (a) Anteroposterior radiograph of the right shin and foot. Talus “wedged” into the ankle syndesmosis (long thick white arrow), fracture of the medial malleolus (short thick white arrow), and proximal fibular fracture (thin white arrow) are seen. (b) Anteroposterior radiograph of the right knee joint. Proximal fibular fracture is indicated with a thin white arrow.

A 30-year-old male patient was admitted to our emergency department with an open fracture of the right ankle. The injury had been a consequence of a crush caused by a fallen freezer. This led to an ankle fracture displacement with disruption of the syndesmosis, trauma to soft tissues and skin, and displacement of the distal end of the tibia through the skin (Figures 1(a) and 1(b)). The three-dimensional CT scanning reconstruction demonstrated displacement of the tibia and wedging of the talus into the distal tibiofibular joint (Figure 1(c)). The injury was rated as Gustilo IIIB type of open fracture [3, 4]. The lateral radiograph of the right shin and foot revealed anterior dislocation of the talus (Figure 2). Furthermore, a fracture of one of the shin bones just distal of the knee joint was also observed (Figure 2). The anteroposterior radiographs of the right shin and knee joint demonstrated fracture dislocation of the right medial malleolus (Figure 3(a)) and fracture dislocation of the proximal third of the fibula (Figures 3(a) and 3(b)). The spiral fracture of the proximal fibula approximated the fractures known as Maisonneuve fractures. Furthermore, the “wedging” of the talus in the ankle syndesmosis (Figure 3(a)) was seen as well. According to the AO-OTA fracture and dislocation classification (https://aotrauma.aofoundation.org and [5]), this patient's diagnosis was categorized as AO-OTA 44B3 and 44C3.

**Figure 4:**
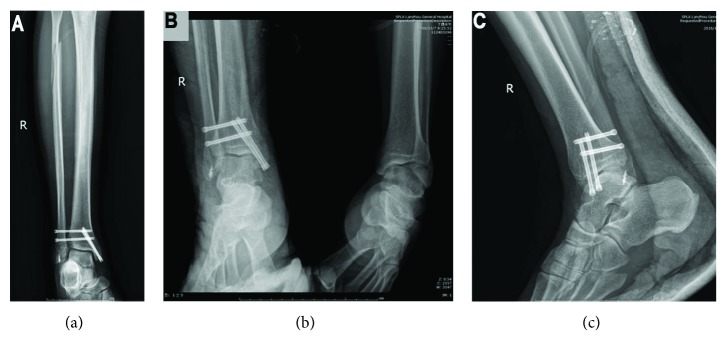
Posttreatment anteroposterior radiographs of the right shin and ankle and lateral radiograph of the right ankle. (a) Anteroposterior radiograph of the right shin and foot. (b) Anteroposterior radiograph of the right ankle. (c) Lateral radiograph of the right ankle.

Emergency surgery began 6 hours after the injury with total debridement, ankle reduction, and suturing. Fracture dislocation of the right medial malleolus was fixed with two cannulated compression screws (Figure 4(a)), while the syndesmosis was supported by two cannulated compression screws (Figures 4(a)–4(c)). The right anterior talofibular ligament was repaired using a suture anchor (Figure 4(a)). A right-side calcaneofibular ligament neoplasty was done as well. After reduction, the proximal fibular fraction did not require treatment beyond immobilization.

**Figure 5:**
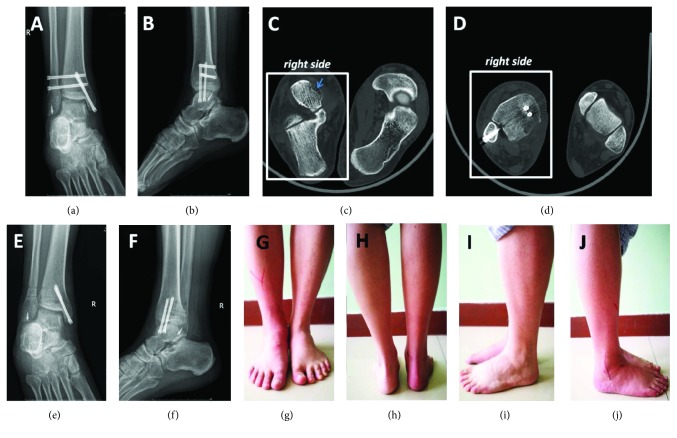
3 months posttreatment: anteroposterior and lateral radiograph images, radiographs after the removal of the syndesmosis screw fixation images, CT scanning of the ankle, and images of the patient standing on his feet. (a) Anteroposterior radiograph of the right ankle. (b) Lateral radiograph of the right ankle. (c) The astragalus contusion is indicated with the arrow. (d) The Dupuytren fracture and dislocation was ameliorated. (e) Anteroposterior radiograph of the right ankle after the removal of the syndesmosis screw fixation images. (f) Lateral radiograph of the right ankle after the removal of the syndesmosis screw fixation images. Images of the patient standing on his feet. (g) Anteroposterior image of the patient standing on his feet. (h) Posteroanterior image of the patient standing on his feet. (i) Lateral image from the left of the patient standing on his feet. (j) Lateral image from the right of the patient standing on his feet.

**Figure 6:**
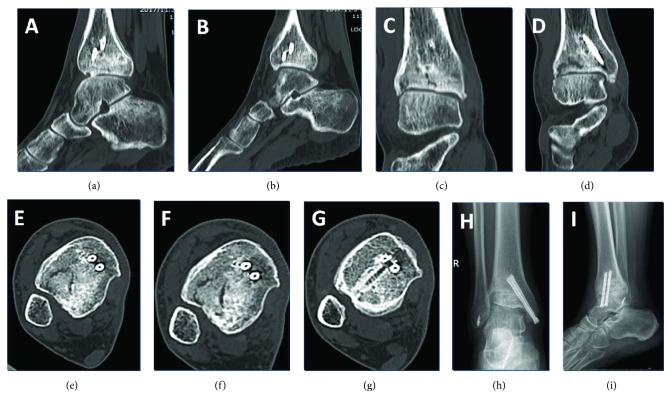
12 months posttreatment: the ankle CT scanning and anteroposterior and lateral radiographs images. (a–f) Ankle CT scanning images of the right ankle. (h) Anteroposterior radiograph of the right ankle. (i) Lateral radiograph of the right ankle.

**Figure 7:**
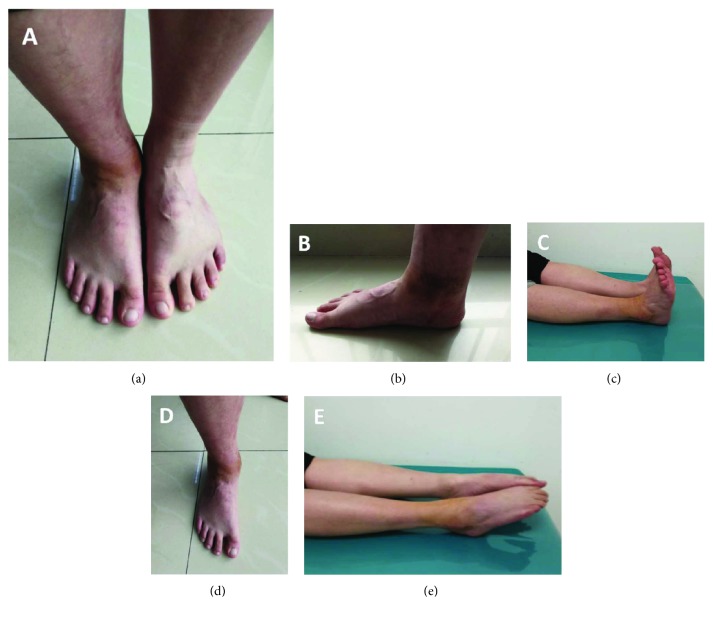
12 months posttreatment: images of the patient standing on his feet. (a) Anteroposterior image of the patient standing on his feet. (b) Posteroanterior image of the patient standing on his feet. (c) Lateral image of the patient standing on his left feet. (d) Lateral image of the patient standing on his right feet. (e) Function of the patient flexion and extension of his feet.

**Figure 8:**
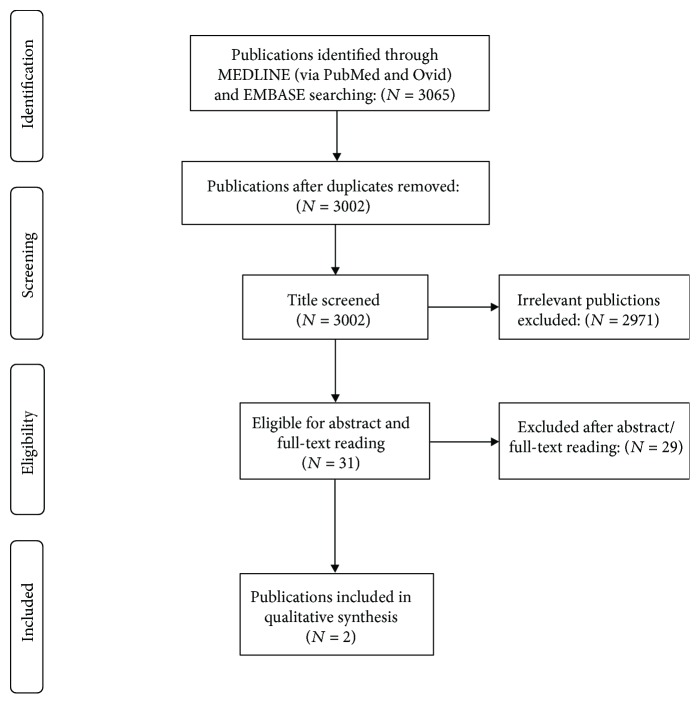
PRISMA diagram of systematic literature review.

Weight-bearing restrictions were applied for the first 3 months posttreatment. Follow-up visits took place 3 and 12 months following the surgery. The 3-month follow-up visit confirmed the healing of the right syndesmosis, and the anteroposterior and lateral radiograph images showed that these fractures' and dislocations' healing process was good (Figures 5(a)–5(d)); the screw fixation supporting the right ankle syndesmosis was removed (Figures 5(e) and 5(f)). At the 12-month follow-up visit after surgery, the radiography examination demonstrated that fractures and dislocations were restored well and healed (Figure 6). Furthermore, rehabilitation of the right ankle function also commenced. While not all sequelae of the ankle fracture disappeared, as, for example, astragalus contusion was still evident, the good functions and satisfied gesture of walking were restored (Figure 7), and the overall outcome was improving after 1-year follow-up. Specifically, the AOFAS Ankle-Hindfoot Scale gradually improved from 40 at one month to 59 at two months to 71 at three months posttreatment, which is comparable to scores observed on follow-up by previous studies [1, 2]. The functionality of the right ankle was restored to a greater degree at the three-month mark (Figure 8). No posttreatment complications reported in previous publications [1, 2] (also discussed below) were present in our patient.

Subsequent to treatment and follow-up of this patient, we wished to conduct a systematic review of the literature published on this fracture. The objective for this literature review was a comparison of the clinical picture and applied treatment between our patient and published reports.

We searched two electronic databases (MEDLINE via PubMed and Ovid, and Embase) using the following keywords: “Logsplitter,” “high-energy,” “ankle injuries,” “ankle fractures,” “fracture dislocation,” and “transsyndesmotic” and Boolean operators “AND”/“OR.” The literature search was limited to publications in the past 10 years but not limited to a particular language.

From the 3065 publications found using the aforementioned keywords, only 31 were found suitable after title screening (Figure 8). The abstract and full-text reading of the selected 31 publications narrowed down the database to the two publications that we had previously known and which are cited throughout this case report [1, 2]. Thereby, to the best of our knowledge, ours is the third presentation on the high-energy transsyndesmotic ankle translocation fracture.

The first of the two suitable publications was a Chinese study (Wang and coauthors [1]). This study retrospectively evaluated a patient registry. The authors of this study selected 41 suitable patients, whose data were separated based on the authors' classification into a typical or atypical high-energy transsyndesmotic ankle translocation fracture. The former was caused by, chiefly, vertical axis stress, whereas the latter was a consequence of rotational stress.

The second publication was an American study (Bible and coauthors [2]). The authors enrolled the total of 23 patients. The study design was a prospective cohort study.

Interestingly, these studies provided descriptions of high-energy transsyndesmotic ankle translocation fractures in the majority of patients (extrusion of the distal tibia, medial malleolus fracture, Volkmann's fracture, etc.; Table 1 and [1, 2]) very similar to the injury sustained by our patient. Furthermore, the treatment applied by these authors and us were also very similar (Table 2 and [1, 2]) and involved an application of one or two syndesmotic screws (> 65%; Table 2 and [1, 2]) and medial malleolus fixation (> 65%; Table 2 and [1, 2]). The most common posttreatment complications were infections (Table 3 and [1, 2]), fracture nonunion, and posttraumatic ankle arthritis (Table 3 and [1, 2]). Among serious complications reported by both publication was one case of a below-knee amputation for recurrent infection [2]. This makes the incidence of 4.3% for this study [2]. Given that the combined patient number is 64 (41 patients in the publication by Wang et al. [1] and 23 patients in the publication by Bible et al. [2]), the incidence in relation to the combined number is ~1.6%.

## 3. Discussion

The ankle joint is a weight-bearing joint, in which the distal tibiofibular joint together with the talus forms a structure that resembles a mortise (a socket or recess used in furniture or door locks). Because of the popularity of the term “mortise,” one of the radiograph positions taken during ankle fractures is called “anteroposterior mortise view.”

The term “syndesmosis” describes the area where distal parts of the tibia and the fibular join. With regard to ankle syndesmosis, the term also includes the supporting ligaments. The ankle syndesmosis is not a typical joint, because it has limited mobility. This is necessary to warrant stability of the joint and permit only specific types of movement. While not frequently, injuries to the syndesmosis do occur, requiring a surgery. However, syndesmosis injuries most commonly occur in the form of a syndesmotic ankle sprain (also called “high-ankle sprain”). These injuries are caused by low-energy impacts, such as during athletic activities [6, 7]. As the name indicates, not always are such injuries associated with bone fractures or dislocations or soft-tissue injury. Often, only ligamentous structures are affected in high-ankle sprains, without associated adverse effects on other structures of this joint [7]. Another difference between high-ankle sprains and the high-energy transsyndesmotic ankle translocation fracture lies in the biomechanics of the injury. The former injury is thought to involve excessive external rotation or eversion or dorsiflection of the foot [8]. In contrast, the clinical case presented above and relevant publications [1, 2] address a different, much rarer type of injury to the ankle syndesmosis.

This injury occurs as a consequence of a high-energy impact to the foot. Most commonly, this fracture is part of polytrauma due to a traffic accident [1, 2]. However, crush by a falling object, like in the clinical case presented here, can also cause the high-energy transsyndesmotic ankle translocation fracture. Traffic accidents, falls, or crushes cause a “wedging” of the talus into the syndesmosis, with subsequent disruption of the syndesmosis, thereby defining the transsyndesmotic character of the injury. Soft tissues are often injured extensively, such as was the case with the aforementioned patient. Because of the tightness of the syndesmosis joint, the wedging of the talus literately forces the other two bones in the joint to move outwards, causing fracture dislocation. Thus, a prior publication on this subject [2] reported that 50% of similar fractures had led to extrusion of the distal tibia through the open wound. This type of injury was also present in our patient.

The surgical and orthopaedic treatment of high-energy transsyndesmotic ankle translocation fractures is different from those applied to more common high-ankle sprains (with or without bone fractures). In the present report, we describe our treatment of a patient with high-energy transsyndesmotic ankle translocation fracture and summarize available literature on this subject. Briefly, a typical patient receives syndesmosis fixation via one or two screws, which is often accompanied by medial malleolus fixation and/or fibula fixation. The prognosis is generally favourable, with infection, nonunion, and posttraumatic arthritis being the most common complications.

## 4. Conclusion

High-energy transsyndesmotic ankle translocation fracture is a rare type of ankle fracture caused by traffic accidents, falls, or crushes by a falling object. The clinical case and systematic review of the literature describe typical clinical manifestations and required treatment.

## Figures and Tables

**Table 1:** Clinical characteristics of high-energy transsyndesmotic ankle fracture dislocation in published reports.

Publication; type of study	*N* of patients	Main causes of the injury (*N*/total *N* [% of total *N*])	Open fracture, *N*/total *N* (% of total *N*)	AO-OTA fracture and dislocation classification (*N*/total *N* [% of total *N*])	Associated fractures and injuries (*N*/total *N* [% of total *N*])
Wang et al. [1]; retrospective review of patient registry	41	(i) Traffic accidents (11/41 [26.8%]) (ii) Falls (from human height) (16/41 [39%]) (iii) Falls from an elevation (10/41 [24.4%]) (iv) Sprains (3/41 [7.3%]) (v) Crush by a falling object (1/41 [2.4%])	16/41 (39%)	(i) 44A (3/41 [7.3%]) (ii) 44B (5/41 [12.2%]) (iii) 44C (33/41 [80.5%])	(i) Distal tibiofibular syndesmotic injury (29/41 [70.7%]) (ii) Triangular ligament injury (18/41 [43.9%]) (iii) Fibula fracture (40/41 [97.6%]) (iv) Medial malleolus fracture (29/41 [70.7%]) (v) Tibial plafond injury (10/41 [24.4%])

Bible et al. [2]; prospective cohort	23	(i) Traffic accidents (10/23 [43.5%]) (ii) Falls from an elevation (10/23 [43.5%]) (iii) (unspecified) crush (3/23 [13%])	12/23 (52.2%)	44B (23/23 [100%])^∗^	(i) Fibula fracture (22/23 [95.6%]) (ii) Medial malleolus fracture (18/23 [78.3%]) (iii) Tibial plafond injury (11/23 [47.8%])

Footnote: ^∗^all patients had been selected based on the 44B classification.

**Table 2:** Treatment applied to high-energy transsyndesmotic ankle fracture dislocation in published reports.

Publication	Syndesmosis fixation (*N*/total *N* [% of total *N*])	Tibial plafond fixation, *N*/total *N* (% of total *N*)	Medial malleolus fixation, *N*/total *N* (% of total *N*)	Other treatments applied
Wang et al. [1]	(i) No syndesmotic screw (12/41 [29.3%]) (ii) One syndesmotic screw (10/41 [24.4%]) (iii) Two syndesmotic screws (17/41 [34.1%]) (iv) Three syndesmotic screws (2/41 [4.9%])	Not indicated	27/41 (65.9%)	(i) Weight-bearing restrictions (ii) Dorsal expansion and plantarflexion starting 2–3 weeks posttreatment

Bible et al. [2]	(i) One syndesmotic screw (4/23 [17.4%]) (ii) Two syndesmotic screws (15/23 [65.2%]) (iii) Three syndesmotic screws (2/23 [8.7%])	8/23 (34.8%)	23/23 (100%)	(i) Ankle immobilization (no further details) (ii) Weight-bearing restriction (no further details)

**Table 3:** Posttreatment follow-up.

Publication	Duration of the follow-up, months	Average AOFAS score at final follow-up visit	Other indicators (*N*/total *N* [% of total *N*]) or mean (SD)	Complications (*N*/total *N* [% of total *N*])
Wang et al. [1]	Up to 12 months ^∗^	>75^∗^	(i) Good Burwell-Charnely radiographic union score (22/41 [53.7%]) (ii) Fair Burwell-Charnely radiographic union score (16/41 [36%]) (iii) Poor Burwell-Charnely radiographic union score (3/41 [7.3%])	(i) Infection (3/41 [7.3%]) (ii) Fracture nonunion (3/41 [7.3%]) (iii) Posttraumatic ankle arthritis (28/41 [68.3%])

Bible et al. [2]	Mean (SD) of 20.6 (6.2) months	Mean (SD) of 67 (26.8)	(i) SMF dysfunction index, mean (SD) of 32.9 (28.6) (ii) SMF bother index, mean (SD) of 34.5 (29.5)	(i) Infection (4/23 [17%]) (ii) Fracture nonunion (4/23 [17%]) (iii) Posttraumatic ankle arthritis (16/23 [69.6%])

Footnote: ^∗^presented separately for patients with typical (caused mainly by vertical axial stress) and atypical (caused mainly by rotational stress) injuries.

## Supplementary Materials

Supplementary Materials Supplementary Material Video 1: 12 months posttreatment: function video of the patient flexion and extension of his feet.
